# Transposed Genes in Arabidopsis Are Often Associated with Flanking Repeats

**DOI:** 10.1371/journal.pgen.1000949

**Published:** 2010-05-13

**Authors:** Margaret R. Woodhouse, Brent Pedersen, Michael Freeling

**Affiliations:** Department of Plant and Microbial Biology, University of California Berkeley, Berkeley, California, United States of America; Stanford University, United States of America

## Abstract

Much of the eukaryotic genome is known to be mobile, largely due to the movement of transposons and other parasitic elements. Recent work in plants and Drosophila suggests that mobility is also a feature of many nontransposon genes and gene families. Indeed, analysis of the Arabidopsis genome suggested that as many as half of all genes had moved to unlinked positions since Arabidopsis diverged from papaya roughly 72 million years ago, and that these mobile genes tend to fall into distinct gene families. However, the mechanism by which single gene transposition occurred was not deduced. By comparing two closely related species, *Arabidopsis thaliana* and *Arabidopsis lyrata*, we sought to determine the nature of gene transposition in Arabidopsis. We found that certain categories of genes are much more likely to have transposed than others, and that many of these transposed genes are flanked by direct repeat sequence that was homologous to sequence within the orthologous target site in *A. lyrata* and which was predominantly genic in identity. We suggest that intrachromosomal recombination between tandemly duplicated sequences, and subsequent insertion of the circular product, is the predominant mechanism of gene transposition.

## Author Summary

Repetitive DNA, such as satellite repeats and transposons, is ubiquitous throughout the genome. Such repeats have been associated with DNA loss, circle formation, and gene transposition in plants and Drosophila. In this work we suggest that, in plants, one mechanism of gene mobility is intrachromosomal recombination via tandem repeats. In addition, we have demonstrated that the classes of genes that tend to form tandem duplications are more likely to have transposed than other gene classes. We conclude that tandem duplications may particularly facilitate gene excision and may also provide targets for gene insertion.

## Introduction

Much of the eukaryotic genome is known to be mobile. This is a characteristic feature of transposable elements, and a large proportion of many eukaryotic genomes are composed of these parasitic elements. However, recent work in plants [1], [2] and Drosophila [3] demonstrates that mobility is also a feature of many non-transposon genes and gene families. An analysis of the Arabidopsis genome suggests that as many as half of all genes had moved to unlinked positions since Arabidopsis diverged from papaya roughly 72 million years ago [4], and that these mobile genes tended to fall into distinct gene families [2]. With the exceptions of unannotated transposons and retroposed genes, the exact mechanism by which single-gene transposition occurred was not deduced, though potential mechanisms include transposon-mediated transduplication or “highjacking” [5], recombination between repeated sequences [6], or nonhomologous end-joining of double-stranded breaks [7]. Unfortunately, ancient gene transposition events, such as those detected in Arabidopsis to date, are an unlikely source of clues because random mutation would be expected to erode all evidence of the mechanism of transposition. In order to detect such evidence, we examined more recent gene transposition events by comparing two relatively closely related (∼5MYA, [8]) Arabidopsis species, *A. thaliana* and *A. lyrata.* In this way we were able to identify a large number of recently transposed *A. thaliana* genes. We found that flanking direct repeats were associated with nearly half of these transposed genes, indicating that these repeats have a role in the process of gene transposition.

## Results

### Transposed genes within Arabidopsis fall into distinct categories

**Figure 1:**
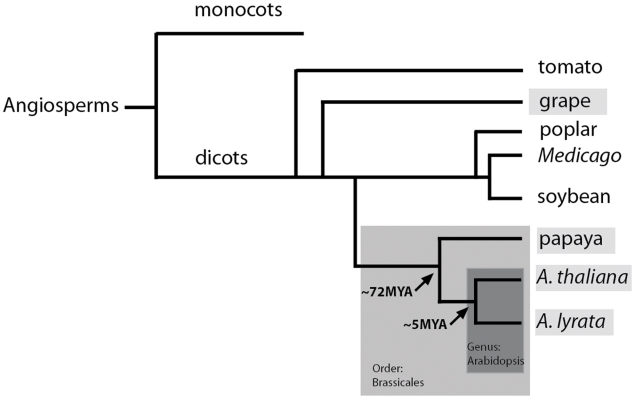
Cladogram of the key species used in this study: Arabidopsis, papaya, and grape. The genus Arabidopsis belongs in the order Brassicales, as does *Carica papaya*. Papaya and Arabidopsis diverged from each other approximately 72MYA. *Arabidopsis thaliana* and *Arabidopsis lyrata* diverged from each other approximately 5MYA.

In order to detect recently transposed nontransposon genes in *A. thaliana*, we used a semi-automated procedure (Methods). Briefly, we automated a procedure we call the flanking gene method, which compares the location of two sequential genes in a region orthologous between two species such that, given genes A and C, if gene B is present between the two genes in one species but not the other, gene B is denoted as a possible transposed gene (as previously described in [2]). This method can identify genes that are present at a given position in *A. thaliana*, but absent at the orthologous position in *A. lyrata.* In order to distinguish between a gain in *A. thaliana* and a loss in *A. lyrata*, each region was compared to the orthologous regions in *Carica papaya* (papaya) and *Vitis vinefera* (grape), two more distantly related species in the rosid clade (Figure 1) (Methods). The absence of a given gene at the syntenous position in both of the outgroups and in *A. lyrata* was interpreted as evidence for insertion in *A. thaliana*; if it was not possible to substantiate the status of the candidate in the outgroup—for example because its expected position in the outgroup was in an unsequenced region—that particular candidate was excluded. We found a total of 420 genes that were present at a given position in *A. thaliana* and absent at the expected position in *A. lyrata.* Analysis using the two outgroup species suggested that that 226 of these genes were new insertions in *A. thaliana* (Table S1). This figure is most conservative, as our methods purposefully discarded questionable data.

As had been observed previously [2], we found that certain gene families are much more likely to have transposed than others (Table 1). Other than genes that encode unknown proteins, *F-box* genes were the most common class of transposed genes (6.2%), followed by *MADS/AGL* genes and LRR-type disease resistance genes (3.5% each), then defensins (2.7%). Defensins, due to their small size and rapidly changing sequences, were mostly undetected in *C. papaya*. Similarly, the same transcription factor gene families that, according to [2], tend *not* to transpose in the period following the divergence of *A. thaliana* and *C. papaya* (e.g. TF-*GRAS* genes, *WRKY* genes, *WD40* genes, and GERMINS), are not found at all within our list of 226 genes that had transposed following the divergence of the *A. thaliana* and *A. lyrata* lineages. Our data suggests that, correcting for divergence time, the rate (in duplication-transpositions/MY) of gene transposition detected when comparing *A. thaliana* and *A. lyrata* (5 MYA) is roughly the same as the rate observed when comparing *A. thaliana* and *C. papaya* (72 MYA) [2]. We can calculate that, since the divergence of *A. thaliana* and *A. lyrata*, gene transposition has occurred approximately once every 22,000 years (226 transposed genes/5 MYA). Altogether, these data suggest that the more recent gene transposition events we detect in *A. thaliana* are representative of transpositions that have been occurring over the past 72 million years in the Arabidopsis lineage.

**Table 1:** The 226 transposed genes in *A. thaliana* and their familial categories.

gene description	number of transposed genes	% of transposed genes
**Other**	100	44.2%
**Unknown**	72	31.9%
**F-box**	13	5.8%
**AGL/MADS box**	8	3.5%
**LRR**	8	3.5%
**Defensins**	6	2.7%
**small secreted cysteine-rich proteins**	4	1.8%
**PR (pathogenesis-related) peptide**	3	1.3%
**DC1-domain containing**	3	1.3%
**nucleic acid binding**	3	1.3%
**beta-galactosidase**	2	0.9%
**tRNA-intron endonuclease**	2	0.9%
**Rapid alkalinization factor (RALF) family protein**	2	0.9%

Represented are genes (besides those described by TAIR as “unknown”) that appear at least twice in our list. All others are put under the category of “Other.” Notice that five of the categories are those that are involved in plant defense (*F-BOX* genes, *LRR* genes, defensins, genes encoding small secreted cysteine-rich proteins, and genes encoding PR peptides).

### Many transposed genes appear to have recently transposed from a parent site

To examine the nature of these gene transpositions in closer detail, we looked for evidence of recent transposition events from a parental or source position. We focused on transposition events that were not the result of retroposition, but rather DNA-level recombination. These are distinguished as transposed sequences that contain intron and/or non-coding flanking sequences that exist in their parental copy. Assuming that gene transposition happens continuously over time, we expect that a recently transposed gene would retain noncoding sequence similarity to its parent if the parent still existed in the genome. We would also expect that only half of all transposed genes would have remaining donor sites within a given genome if the donor site and the transposed gene were unlinked, as, once transposition occurs, the donor site is then heterozygous for gene in question, and the donor site may be lost via segregation. However, the ratio of identifiable donor sites may be even less than half if the transposition event in question had been relatively ancient, and the noncoding sequences for the donor site and transposed gene had significantly diverged. We felt that the most recent transposition events would also retain evidence of their mechanism of transposition, and we focused on those. To do this, we looked for an unlinked paralog that had sequence similarity with the transposed genes higher than 75% identity across at least 50 base pairs of *noncoding* sequence. These criteria were used in order to restrict the level of detection to genes that had only recently transposed. Of the 126 transposed genes we examined manually (excluding the “unknown” genes), 106 of our transposed genes did not have a best hit with noncoding sequence similarity greater than or equal to 75%/50 bp. However, 25 (19.8%) had a best hit whose noncoding sequence fit the above criteria, consistent with being relatively recent transposition events, and making them candidate source genes (Table 2, Table S2). In 60% (15/25) of such cases, the parental gene was in a position syntenous in *A. lyrata*, suggesting that the parental gene itself had not transposed from another position within the last 5 MY. As a control, we also examined 102 genes that had not transposed since the divergence of *A. thaliana* and *A. lyrata* (Methods). None of these genes had a best hit whose noncoding sequence similarity was above 75% identity over 50 bp (Table 2, Table S3).

**Table 2:** Sequence similarity with best blastn hit and frequency of flanking repeats in transposed versus nontransposed genes.

	Number of genes examined	Number with flanking repeats >15 bp	% with flanking repeats >15 bp	number with flanking repeats >30 bp	%with flanking repeats >30 bp	Number of genes with a best hit whose noncoding sequence >75%ID/50 bp	% of genes with a best hit whose noncoding sequence >75%ID/50 bp
**Transposed**	126	22	17.5%	18	14.3%	25	19.8%
**Not transposed**	102	6	5.9%	2	2.0%	0	0.0%

Comparing frequency of genes with significant flanking repeats and noncoding best hits between transposed and nontransposed genes. None of the 102 nontransposed genes in our study had a best hit with noncoding sequence greater than 75% identity over 50 bp, as opposed to 19.8% of transposed genes that did have a best hit that fit that criteria.

### Flanking direct repeats are associated with transposed genes

Next, we examined our transposed genes for signatures of the transposition mechanism. In particular, we searched for the presence of direct repeats flanking the transposed sequence because such repeats have already been shown to be associated with indels in Arabidopsis (though the absence of an outgroup prevented the distinction between insertion and deletions) [9]. In addition, whole-gene transposition in Drosophila [3] has also been associated with direct repeats of highly repetitive DNA. To look for flanking direct repeats around our transposed genes, we used the genome visualization platform GEvo to visually compare the 5′ region ∼500 bp upstream of our target sequence to the sequence ∼500 bp downstream of its 3′ region (Methods). We limited our search to BLAST hits that were greater than or equal to the e-value of a 15/15 bp exact match, and excluded simple sequences. Using these criteria, 17% (22/126) of our total transposed genes had flanking repeats greater than 15 bp (Table 3). However, when we enriched for transposed genes that had an identifiable parental site, 44% (11/25) had flanking direct repeats equal to or greater than 15 bp in length. In contrast, only 5.9% (6/102) of the control, nontransposed genes had flanking repeats greater than 15 bp (Table 3) (p-value 0.00025). The difference was even more dramatic for longer repeats: 36% (9/25) of parental-site transposed genes had flanking repeat sequence over 30 bp in length, versus only 2% (2/102) of the control genes (p-value 0.000051).

**Table 3:** Flanking repeat frequency for transposed genes with a best noncoding hit versus non-transposed genes.

	Number of genes examined	Number with flanking repeats >15 bp	Number with flanking repeats >30 bp	% with flanking repeats >15 bp	% with flanking repeats >30 bp
**Transposed/duplicated**	25	11	9	44.0%	36.0%
**not transposed**	102	6	2	5.9%	2.0%

Incidence of flanking repeats is much higher for duplicated/transposed genes than for transposed genes overall (Table 2) (44% vs 17.5% respectively) and is higher than for all nontransposed genes examined (5.9%).

**Figure 2:**
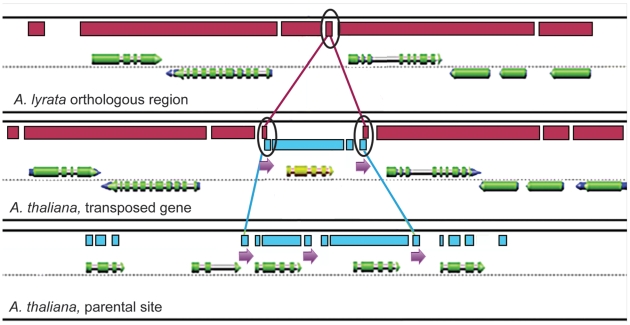
Flanking direct repeats around transposed genes in Arabidopsis. A model of a transposed gene surrounded by flanking repeats, based on the graphics of our genome visualization platform GEvo (http://synteny.cnr.berkeley.edu/CoGe/GEvo.pl). The middle panel represents the transposed gene (yellow), the top panel represents the sequence *in A. lyrata* that is the orthologous region to the transposed gene locus, and the bottom panel represents the parental site in *A. thaliana* from which the mobile gene transposed. The long red rectangles above the genes in the top and middle panels represent orthologous sequence between *A. thaliana* and *A. lyrata*; the long blue rectangles in the middle and bottom panels represent sequence similarity between the transposed *A. thaliana* gene and the parent gene of origin. Purple arrows beneath the sequence represent direct repeats in *A. thaliana* and the sequence homology between the direct repeats in *A. lyrata.* Circled are the repeated sequences that are similar among the *A. thaliana* parental site, the transposed gene, and the target site in *A. lyrata* (note that the sequence only appears once in the *A. lyrata* target site). This model suggests that the repeat sequence flanking the transposed gene (circled rectangles, center panel) is a chimera between the donor site repeat sequence and the *A. lyrata* target site (also see Figure 3B). Notice that the parent site is a tandem duplication.

Upon closer examination of the nine transposed genes whose flanking repeats were greater than 30 bp, we found that in six cases, the flanking repeat sequence was detected at the parent site at least once (Table 4, Figure 2, Figure S1). Repeat carryover from a parent sequence had been associated with transposed genes in Drosophila [3], suggesting a flanking-repeat excision model. Two of our nine long-direct-repeat genes had inverted repeats associated with them as well as direct repeats (AT5G10330 and AT1G49715, Table 4). Inverted repeats are a hallmark of DNA transposons that are known to “highjack” foreign DNA sequence [5]. In fact, one of our transposed genes in this study, AT1G49715 was a PACK MULE, with characteristically long terminal inverted repeats flanked by 10-bp target-site duplications. Of the unknown genes excluded from this study, we found at least five genes that had clearly been captured by a transposon-like mechanism, based on the fact that were not transcribed, only a portion of them transposed from their putative donor site, and they were flanked by either terminal inverted repeats (TIRs) or long terminal repeats (LTRs) (data not shown). In short, we were able to detect transposons and transposons with genic insertions, so these did not confuse our study of the transposition of more typical genes.

**Table 4:** Transposed-duplicated genes and their best blastn hits, with flanking sequence >30 bp in length.

GENE ID	TAIR v. 9 gene description	best hit in At	Repeat size (bp)	repeat type	repeat sequence is present in A. lyrata orthologous region	repeat sequence identity	repeat sequence is present in parent	parent sequence is a tandem duplication
AT5G10330	histidinol-phosphate aminotransferase	AT1G71920	193	direct + inverted	no	inverted repeat is an unknown gene	yes (direct repeat)	no
AT1G49715	defensin-like (DEFL) family protein	AT1G73607	550	direct + inverted	no	AT-rich sequence	yes (direct repeat)	yes
AT2G34290	protein kinase family protein	AT5G27790	200	direct	yes	similar to dehydration-responsive protein	yes	no
AT2G26010	PR (pathogenesis-related) protein	AT5G44430	69	direct	yes	within transposed sequence	yes	yes
AT2G16930	ribosomal protein L27 family protein	AT5G15220	72	direct	yes	RNA recognition motif (RRM)-containing protein	no	no
AT3G10845	RNA recognition motif (RRM)-containing protein	AT5G41690	79	direct	yes	hydroxyacylglutathione hydrolase	no	no
AT4G01640	F-box associated type 1	AT2G34280	234	direct	yes	unknown protein	no	no
AT5G01080	beta-galactosidase	AT1G30784	34	direct	yes	unknown protein (AT5G01370) mRNA	yes	no
AT1G23810	paired amphipathic helix repeat-containing protein	AT1G27270	80	direct	no	unknown; within transposed sequence	yes	yes

Flankers for five of the nine genes have genic content. Three of these mobile genes appear to have transposed from a tandem repeat. The flanking sequence for five of the genes appears in the orthologous region in *A. lyrata*.

**Figure 3:**
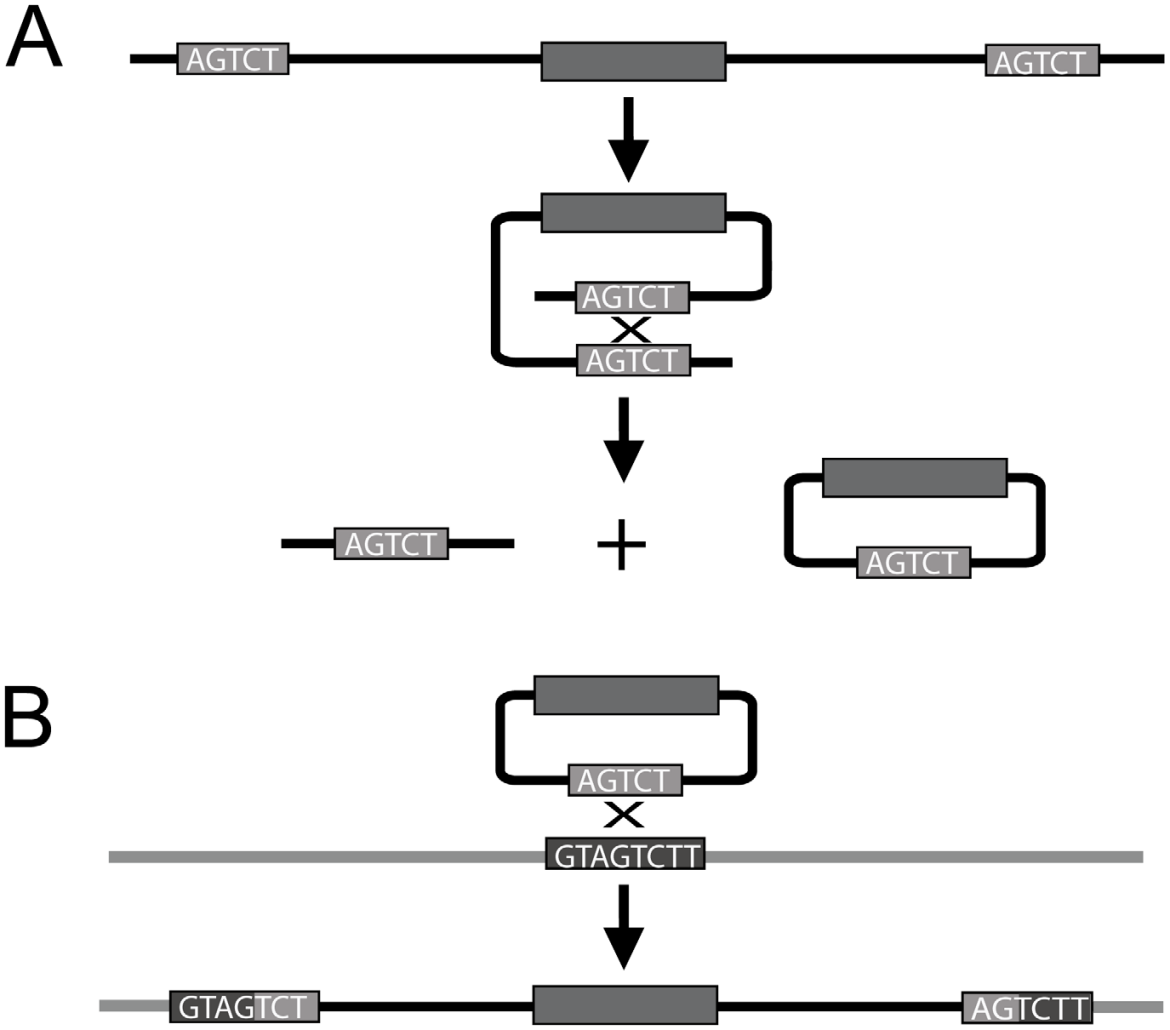
Diagram depicting intrachromosomal recombination and insertion via recombination of homologous sequence. A gene (grey rectangle) bracketed by direct repeats (A) may form a circle, which then might be integrated elsewhere in the genome if the target region contains sequence similarity to the flanking repeats (B). In the case of this figure, the slight dissimilarity between the repeat sequence of the flanking repeats surrounding the transposed gene and the homologous sequence of the target sequence, may give rise to flanking sequences at the target site that no longer share sequence similarity.

Unexpectedly, in five of the transposed genes with long flanking repeats, the sequence of the repeat could be found at the orthologous site in *A. lyrata* (AT2G34290, AT2G26010, AT3G10845, AT4G01640, and AT2G16930, Table 4). This *A. lyrata* sequence could well correspond to the recombination site for the insertion in *A. thaliana*. In addition, of these five genes, three (AT2G34290, AT2G26010, and AT5G01080) were flanked by repeat sequence that could also be found at the parental site at least once, as though this repeat sequence were a source for intrachromosomal recombination *out* of the site of origin as well as recombination *into* the target site orthologous to the *A. lyrata* homologous sequence (as illustrated in Figure 3). Notably, the repeats surrounding two of these genes (AT2G26010 and AT5G01080) were chimeric between the original flanking sequence and the *A. lyrata* homeologous target sequence, as demonstrated in Figure 3B. Additionally, among the transposed genes in our study that did *not* have an identifiable parent source or donor site, nine of these unpaired, transposed genes also had flanking repeat sequence greater than 30 bp in length within 500 bp of the coding region. In eight of these cases, the flanking repeat sequence was found at the orthologous target region in *A. lyrata* (Table 5). Therefore, among the eighteen total transposed genes that had flanking repeats over 30 bp in length, thirteen (72%) of them had repeat sequence that corresponded to the orthologous site in *A. lyrata*.

**Table 5:** Transposed Arabidopsis genes without a likely “parent” gene.

GENE ID	TAIR v. 9 gene description	Repeat size (bp)	repeat type	repeat sequence in A. lyrata orthologous region	repeat sequence identity
AT2G25450	encodes a protein whose sequence is similar to ACC oxidase	120	direct	no	3′ of the gene
AT3G10430	F-box family protein	31	direct	yes	unknown protein (AT1G18740) mRNA
AT3G18120	F-box family protein-related	30	direct	yes	receptor for activated C kinase
AT4G26870	aspartyl-tRNA synthetase, putative	287	direct	yes	3′ of the gene
AT4G33900	kelch repeat-containing F-box family protein	36	direct	yes	unknown protein
AT5G12280	RNA binding	66	direct	yes	unknown protein
AT5G52090	tRNA-splicing endonuclease positive effector-related	116	direct	yes	DRE/CRT-binding factor 1
AT5G57890	anthranilate synthase beta subunit, putative	35	direct	yes	F-box protein
AT5G58120	disease resistance protein (TIR-NBS-LRR class), putative	80	direct	yes	ATPase activator/chaperone binding, RNA recognition motif (RRM)-containing protein

Eight out of the nine genes have flanking sequence which corresponds to sequence in the orthologous region of *A. lyrata*. The sequence identity of all repeats is gene-like, not repetitive or transposon-associated.

### The gene classes that tend to transpose are associated with flanking direct repeats whose sequence is genic, not transposon

Previous work in Drosophila found that the sequence identity of repeats surrounding transposed genes usually comprised transposon sequence [3], and an argument was made suggesting that transposon sequence distributed randomly within the genome could facilitate gene transposition by ectopic recombination. However, when we examined the sequences of the repeats flanking our transposed genes, we generally found these repeats to be made up of host non-transposon sequence of varying kinds, including what appear to be the remnants of fractionated genes (Table 4, Table 5).

We decided to examine all genes in the *A. thaliana* genome to determine whether the gene classes that tended to transpose were more likely to be flanked by direct repeats, and if so, what the identity of the sequence of those direct repeats were. We made a prediction that the gene classes that tended to transpose would also tend to be flanked by direct repeats; such flanking repeats would endow these gene classes with a propensity for excision and insertion via ectopic recombination.

Using an automated blast search with the parameters described in Methods, we retrieved 1088 genes, including pseudogenes, that were identified as having flanking direct repeats according to the algorithm. These included genes that had not transposed in the 5 MY since the *A. thaliana/A. lyrata* split. When we examined the classes of genes that tended to have these flanking repeats, we found that, aside from unknown genes, F-box genes were the highest represented as being flanked by direct repeats (4.5%,) closely followed by pseudogenes, then defensins and LRR genes (2.8% and 2.2%, respectively) (Table 6). These percentages are proportional to the percentages that these gene families make up in transposed genes (Table 1). Most notably, these three gene classes are also those that tend to either form, or insert into (interrupt), tandem duplications. Indeed, in 80% of the cases examined, the sequence identity of the flanking repeats is genic, and in the cases of F-box genes and LRR genes, the sequence is similar to the gene itself 40% of the time, as though the flanking repeats are remnants of a tandem duplication.

**Table 6:** Gene families flanked by repeats.

Gene description	number of genes with flanking repeats	% of genes with flanking repeats
other	501	46.0%
unknown	203	18.7%
F-box	49	4.5%
pseudogene	33	3.0%
defensins	30	2.8%
LRR	24	2.2%
zinc finger family protein	19	1.7%
small secreted cystiene-rich proteins	18	1.7%
protein kinase family protein	15	1.4%
DC1 domain-containing protein	15	1.4%
receptor-like protein kinase-related	14	1.3%
ECA1 gametogenesis related family protein	14	1.3%
serine carboxypeptidase-like	12	1.1%
ubiquitin family protein	12	1.1%
Receptor Like Protein	11	1.0%
thionins	9	0.8%
meprin and TRAF homology domain-containing protein/MATH domain-containing protein	9	0.8%
PR (pathogenesis-related) peptide	8	0.7%
putative cytochrome P450	8	0.7%
ATP binding	8	0.7%
protease inhibitor/seed storage/lipid transfer protein (LTP)	8	0.7%
UDP-GLUCOSYL TRANSFERASE	8	0.7%
B3	7	0.6%
invertase/pectin methylesterase inhibitor family protein	7	0.6%
glycoside hydrolase family	7	0.6%
self-incompatibility protein-related	7	0.6%
auxin-responsive	6	0.6%
Potential natural antisense gene	6	0.6%
MADS/AGL	5	0.5%
nucleic acid binding/zinc ion binding	5	0.5%
SKP1-LIKE	5	0.5%
pentatricopeptide (PPR) repeat-containing protein	5	0.5%

Only gene families with five or more representatives were included. As with classes of transposed genes in general, F-box genes, LRR genes, and defensins are among the highest represented as having flanking repeats.

## Discussion

Repeat sequences in general are unstable regions of the genome due to their potential for unequal crossing over, intrachromosomal crossing over, and ectopic recombination [10], [11]. Intrachromosomal recombination between flanking repeats—the sort of event that generates a circular fragment—has been associated in plants and Drosophila with small deletions within transposons. Flanking repeats have also been associated with indels in Arabidopsis, but insertions were not distinguished from deletions [9]. Yang et al. have shown that whole-gene transpositions in Drosophila are primarily associated with 44- to 433-bp flanking repeats of transposable element sequences [3]. Our results in Arabidopsis are similar to those in Drosophila, but our flanking repeats generally derive from host non-transposon sequence.

Forty-four percent of our transposed genes that had an identifiable donor site had (detectable by our Methods) flanking repeats at their insertion site, and thirty-six percent had flanking repeats greater than 30 bp in length. Most transposed genes (with or without an identifiable donor site) with repeats greater than 30 bp were flanked by direct repeat sequence that was homologous to sequence within the orthologous target site in *A. lyrata*. One explanation for this pattern might be that the target site was duplicated following the insertion in *A. thaliana*; such long target-site duplications have been observed in *H. pylori* genomes after gene transposition [12], and in humans due to retroviral insertion [13]. However, there are simpler explanations, as follows.

In the majority of cases, the repeat sequence flanking the transposed gene consisted of non-transposon host sequence, particularly sequence that corresponds to genic sequence, suggesting that the insertion site had once been part of a tandem repeat, or that the transposed gene had originally resided in a tandem repeat; indeed, the parental gene for three of the nine donor-site transposed genes was part of a tandem duplication (Table 4). Previous work in our lab has demonstrated that mobile genes—transposon and nontransposon alike—in Arabidopsis are often found within tandem repeats from unrelated families [2]; these were called “interruptor” genes. In addition, genes that tend to have duplicated in tandem—such as *F-box* genes, *DEFENSINS*, and *NB-LRR*-type disease-resistance genes—also tend to be those that transpose [2], [14]. Findings by Zhou and coworkers suggested that, in Drosophila, tandem repeats may precede DNA transposition [15]. Other researchers in Drosophila had found the presence of chimeric genes within tandem arrays, and postulated that the mechanism may be via large-loop mismatch repair, where a portion of the DNA sequence between duplicated genes is excised [16]. Further, Cohen and coworkers have demonstrated that tandem duplications are associated with the formation of circles in plants [17] and Drosophila [18], where it is thought that a gene that is part of a tandem duplication can excise by intrachromosomal recombination via the repetitive sequence surrounding it, then potentially integrate into a new region containing sequence homologous to the flanking repeats. In fact, in three of our transposed genes (AT2G34290, AT5G15220, and AT2G26010), a portion of the flanking sequence was found in both the *A. lyrata* orthologous target sequence *and* the parental sequence, suggesting the possibility that this same repeat sequence facilitated intrachromosomal recombination and excision from the parental site, then insertion into the homologous target site. Alternatively, ectopic recombination between unlinked sites (say, between one tandem array of a given gene type and another tandem array of the same or similar gene type) may play a role in gene movement; indeed, one hypothesis for the formation of chimeric disease-resistance genes in plants is via ectopic recombination [19].

In over half the cases where a transposed gene had an identifiable parent, no evidence of repeats were found flanking the transposed gene. There are several explanations for this. For instance, the parameters of our experimental design (15/15 exact match) would rule out repeats that were smaller than 15 base-pairs, or repeats that had degenerated beyond what our algorithm could detect. Indeed, in some cases, when we more closely examined the sequence flanking some of the transposed genes which, according to our parameters, were deemed devoid of repeats, we did observe the traces of direct repeats whose sequence similarity would not have been detected by our criteria (data not shown). This is also observed at the parent sites for our transposed genes. In other cases, the donor site contained only one copy of the flanking repeat found at the transposed site. Again, degeneration of the second repeat might have occurred, or the repeat might have been removed entirely via a deletion event [9], [10]. Alternately, recombination with the homologous sequence at the new site might have created chimeric flanking sequences (as illustrated in Figure 2 and Figure 3B). For instance, evidence of flanking direct repeats that are chimeric between the target sequence and the donor repeat sequence is observed in AT2G26010, and AT5G01080, as previously discussed. Particularly after sequence divergence, the original flanking sequence in some instances may not manifest as identifiable direct repeats surrounding the transposed sequence.

Tandemly repeated genes and genes flanked by tandem repeats are a special case because they are regions that are particularly prone to intrachromosomal recombination and the associated circular fragment. As diagrammed in Figure 3, if a sequence in a circular fragment is homologous to a sequence elsewhere in the genome, insertions resulting in flanking repeats are possible. However, reinsertion is likely to be an exceptional event. In most cases, we would expect that the excised circle would simply be lost and thus, these recombination events would result in a net reduction of genomic DNA content. We suggest that this process is the way plants [10] and Drosophila [20] have countered transposon buildup. The gene loss mechanism in plants and other clades able to carry high mutational load is likely to involve the generation of circular fragments of chromosome, and, thus, the potential for transposition.

We calculate the rate of gene transposition as being once every 22,000 years, and hypothesize that the rate of transposition is steady and not punctuated, as the degree of sequence similarity in noncoding sequence between donor and transposed sites varies from 100% identity over all noncoding sequence to 70% identity/50 bp at the very most (Table S2), suggesting that transposition has occurred at different points throughout evolutionary time. This is also consistent with our observation that gene transposition following the divergence of *A. lyrata* from *A. thaliana* has occurred at roughly the same rate as it had occurred since following the divergence of *A. thaliana* from papaya.

Repeats, from a few base-pairs in length to hundreds of base-pairs, are ubiquitous throughout the genome. Via ectopic recombination, these repeats permit the deletion or insertion of a fragment of DNA as small as a few base-pairs, or as large as an entire gene. It seems likely that the genome's potential to re-shape itself in novel ways is built in, not via any special mechanism or adaptation, but as a passive by-product (a spandrel) of the genome's architecture. Obviously the presence of thousands of transposable elements within any genome is a source of recombination; but tandem duplications clearly can play a role as well, particularly at the genic level. Indeed, the fact that many of the repeats surrounding transposed genes in Arabidopsis were associated with what appear to be duplicated gene fragments, and the fact that transposed genes tend to not only form tandem repeats themselves, but insert into tandem repeats, suggests that tandem duplications may particularly facilitate gene excision as well as provide targets for gene insertion.

## Methods

### The list of 420 transposed genes in *A. thaliana*

Automation: Genomes of *A. thaliana* (TAIR masked repeats 50x, v7) and *A. lyrata* (JGI unmasked, v1, sequenced by the Weigel lab http://www.phytozome.net/alyrata) are co-annotated to each other, so that features (files of the annotations used are available here http://syntelog.com/t/gray_paper_methods/) that are annotated in one but not the other become new features in their respective genomes (this co-annotation step is performed by software available from our source-code repository: http://bpbio.googlecode.com/svn/trunk/co-anno). New fasta files are created from the original genomic features and the new ones discovered via co-annotation. The fasta file from *A. thaliana* is blasted against the fasta file from *A. lyrata* using blastn/e-value of 0.1/word size 7. The blast is then “dissolved” by combining small hits to the same gene pairs into single hits, then keeping only those merged hits with a sum length greater than or equal to 96.

The search for transposed genes is performed using the aforementioned dissolved pairs, such that for each gene in a pair, the algorithm is extended out three genes in either direction, creating a list of (gene +3+3)*2 = 14 genes; next, for that group of genes, the flanking gene method is used to find transposed genes in both query and subject by converting gene names to integer positions to get a list of query-subject pairs; for instance: [(1, 123), (2, 125)]. From this, simple addition and matching is used to find any genes that are “missing,” e.g. the gene at position 124 above is unaccounted for, and flanked by consecutive genes 1, 2 from the ortholog. The integer positions are then converted back to gene names, and the transposed gene and the orthologs of its flankers are reported.

Each putative transposed gene is then checked to see whether it lies within a region in a manually generated list of orthologous regions, since a genes in non-orthologous regions cannot be verified as transposed they are discarded. Each putative transposed gene is then blasted against the non-coding sequence in the orthologous flanking region to determine whether it actually appears in the ancestral position even though it is not annotated as such. No BLAST to the transposed gene over 15 bp can be observed between the flankers in *A. lyrata*, otherwise it might just be gene loss in *A. lyrata* (hits_within_0<15). Each putatively transposed gene must be flanked by two genes that are not identical to each other in *A. thaliana* such that *A. lyrata* flanker_1≠ flanker_2. If it fails this (e.g. *A. lyrata* flanker_1 =  flanker_2) then it is designated an “interrupter” or “I” gene. Any pseudogene, or any gene that has been identified as repetitive or transposable element sequence of any kind by the Arabidopsis genome database TAIR (http://www.arabidopsis.org/), is removed from the list.

Proofing: Once the list of putative transposed genes is at hand, each gene is visualized in our gene visualization platform GEvo (http://synteny.cnr.berkeley.edu/CoGe/GEvo.pl) via a link provided in the output containing the CoGe identifier of the putative transposed gene as well as the identifier for one of the *A. lyrata* flankers.

### Using the orthologous groups *C. papaya V. vitifera* to confirm gene transposition in *A. thaliana*

Using blastn parameters of word-size 7 and filtered to exclude simple sequences, each transposed gene was blasted to *C. papaya* (University of Hawaii v0.4, masked repeats 50x) and *V. vitifera* (French National Sequence Center v1, masked repeats 50x) to verify the presence of the gene within the genome, masking all non-cd sequence. We then masked the transposed gene itself and blasted 15 Kb on either side of the gene to *C. papaya* and *V. vitifera* to find the syntenous regions.

If the gene itself was not found in either papaya or grape, it was labeled “No gene hit” and was discarded from our list. If there was a gene hit to papaya and grape, but the surrounding region of *A. thaliana* does not hit in the same region in papaya and grape, the gene was labeled “No hits with gene and buffer in same region” and was considered transposed. If there was a hit to the same region of papaya and grape in both the buffer and the gene itself, it was considered to be in the ancestral position and was discarded as not being a true transposition. Each gene had to have a hit in both papaya and grape, and each gene had to be in a nonancestral position in both papaya and grape, otherwise it was discarded from our list. This procedure left us with the 226 confirmed transposed genes that were the basis of this research (Table S1).

### Finding duplicates of the transposed genes

The genomic sequence (including non-coding sequence) of all genes considered true transpositions in our list were then blasted to the *A. thaliana* genome to find the best hit outside of itself. We then examined the best hit to see whether it had sequence similarity with the transposed gene higher than 75% identity across at least 50 base pairs of noncoding sequence. If the best hit fit these criteria, it was deemed to be a putative parental site from which the transposed gene had duplicated (Tables S2, S3).

### Finding flanking repeats surrounding the duplicated/transposed genes

We used our genome visualization platform GEvo to visually compare the 5′ region ∼500 bp upstream of our target gene to the sequence ∼500 bp downstream of its 3′ region. We limited our search to BLAST hits that were greater than or equal to the e-value of a 15/15 bp exact match, and excluded simple sequences (Tables S2, S3). The 102 non-transposed genes that were selected for the control were genes chosen from specific gene families that had been confirmed by both [2] and this particular study as being underrepresented for gene transposition. Since this portion of the study was performed manually we restricted our analysis to only 102 genes, similar in number to the total number of transposed genes examined for parental sites and flanking repeats (126).

### Automation of flanking repeat discovery around genes in *A. thaliana*

We wrote a program using blastn, word-size 7, with BLAST hits that were greater than or equal to the e-value of a 15/15 exact match, that would look for repeats between 30–400 bp shared between the 2 kb region up and downstream of coding sequence of each gene (Table S4).

## Supporting Information

Figure S1 Flanking direct repeats around transposed genes in Arabidopsis. Three examples of transposed genes surrounded by flanking repeats. For each figure, the middle panel represents the transposed gene (yellow), the top panel represents the sequence in *A. lyrata* that is the orthologous region to the transposed gene locus, and the bottom panel represents the presumptive locus in *A. thaliana* from which the mobile gene transposed (yellow gene is the best blast hit). Pink rectangles represent orthologous sequence between *A. thaliana* and *A. lyrata*; green rectangles represent sequence similarity between *A. thaliana* and *A. lyrata*. Purple squares represent direct repeats. Blue squares represent inverted repeats. These figures were created using our genome visualization platform GEvo (http://synteny.cnr.berkeley.edu/CoGe/GEvo.pl), using the parameters BlastN, with a spike-length of 25 (A–B) or BlastZ (C). (A) AT1G23810. Direct repeats flank the transposed sequence (center), and occur once in the parent site (bottom). Notice that the parent site is a tandem duplication. Also notice that the transposed sequence includes the introns and the regions outside of the coding sequence, suggesting that the transposition was DNA-based and not an RNA-intermediate retroposition. (B) AT1G49715. A PACK MULE that is flanked by inverted repeats (blue). Sequence similarity between the direct repeats of the transposed gene and the parent gene are not shown here due to the parameters used to create the image for this Figure. (C) AT3G10845. Transposed gene is flanked by a direct repeat whose sequence occurs as a singlet in the *A. lyrata* orthologous site (top). Notice that the repeat sequence corresponds to the 3′ untranslated region of the gene adjacent to the transposed gene.
(9.61 MB TIF)

Table S1 The 226 transposed genes used in our study. These are genes that have been confirmed as present at their site due to insertion in *A. thaliana* rather than loss in *A. lyrata* by using the two outgroups *C. papaya* and *V. vitifera* for comparison. Columns I and J provide a link to our GEvo platform to visualize the blast hits of the orthologous regions in *A. thaliana* and *A. lyrata* (for At v7 and At v9, respectively).
(0.72 MB XLS)

Table S2 The best hits of all 126 transposed genes (excluding unknown genes). Column Q (Best hit alignment) provides a link to our GEvo platform to visualize the blast hits between the transposed gene and its best hit (if applicable). Column R (Flanking repeat alignment) provides a GEvo link to the flanking repeats surrounding the transposed gene (if any).
(0.40 MB XLS)

Table S3 The best hits of 102 non-transposed genes (if any). GEvo alignment (Best hit alignment) Column M, GEvo flanking repeat alignment (Flanking repeat alignment) Column N.
(0.32 MB XLS)

Table S4 All genes within the *A. thaliana* genome that are flanked by direct repeats, according to our algorithm described in Methods. The GEvo link is provided in Column K.
(1.67 MB XLS)
