# Supplementary figures and images for: Transposed Genes in Arabidopsis Are Often Associated with Flanking Repeats
Source: PLoS Genet. 2010 May 13;6(5):e1000949. doi: 10.1371/journal.pgen.1000949 (PMC2869330; doi:10.1371/journal.pgen.1000949)

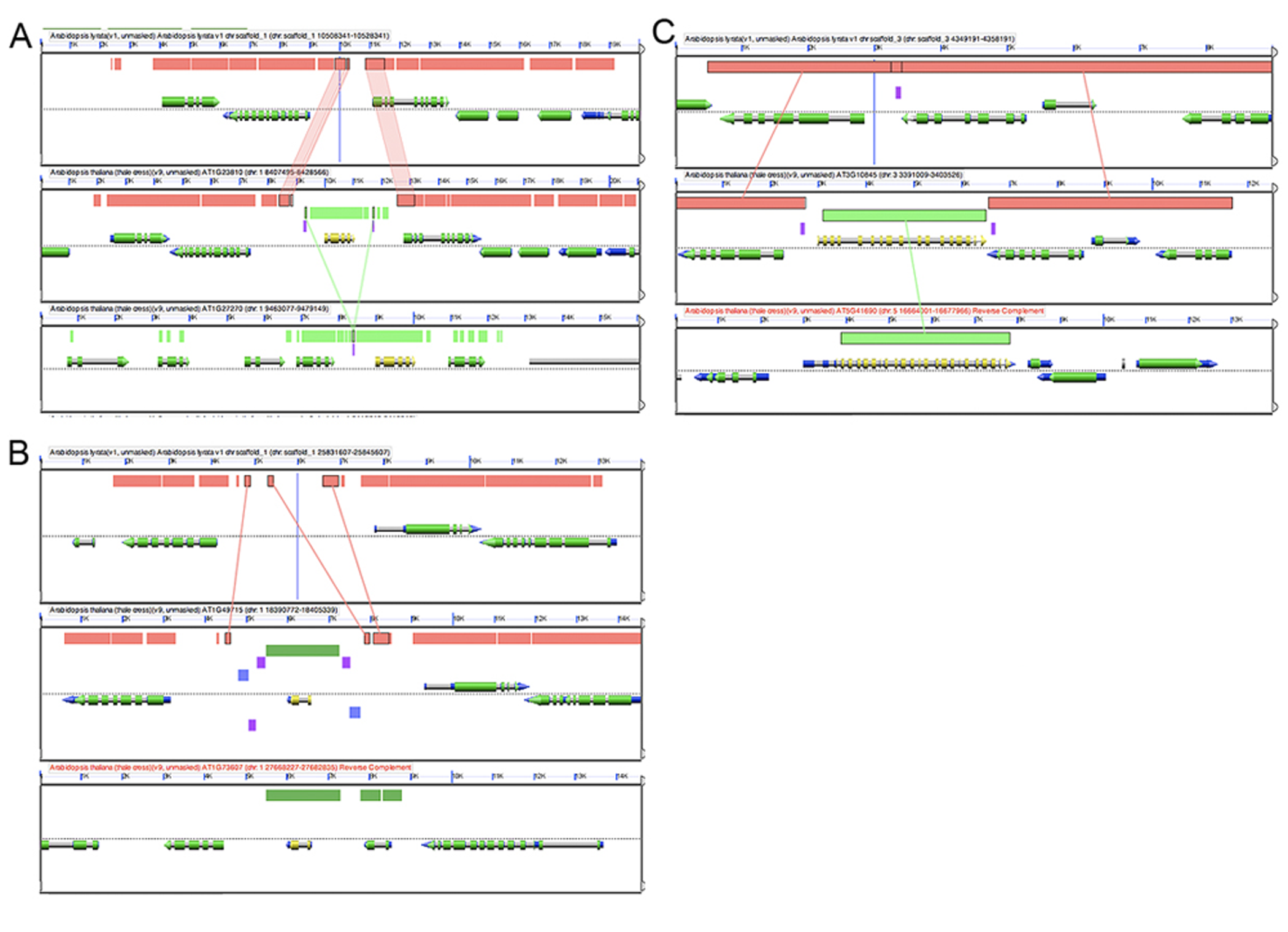

Supplement: Figure S1 — Flanking direct repeats around transposed genes in Arabidopsis. Three examples of transposed genes surrounded by flanking repeats. For each figure, the middle panel represents the transposed gene (yellow), the top panel represents the sequence in A. lyrata that is the orthologous region to the transposed gene locus, and the bottom panel represents the presumptive locus in A. thaliana from which the mobile gene transposed (yellow gene is the best blast hit). Pink rectangles represent orthologous sequence between A. thaliana and A. lyrata; green rectangles represent sequence similarity between A. thaliana and A. lyrata. Purple squares represent direct repeats. Blue squares represent inverted repeats. These figures were created using our genome visualization platform GEvo (http://synteny.cnr.berkeley.edu/CoGe/GEvo.pl), using the parameters BlastN, with a spike-length of 25 (A–B) or BlastZ (C). (A) AT1G23810. Direct repeats flank the transposed sequence (center), and occur once in the parent site (bottom). Notice that the parent site is a tandem duplication. Also notice that the transposed sequence includes the introns and the regions outside of the coding sequence, suggesting that the transposition was DNA-based and not an RNA-intermediate retroposition. (B) AT1G49715. A PACK MULE that is flanked by inverted repeats (blue). Sequence similarity between the direct repeats of the transposed gene and the parent gene are not shown here due to the parameters used to create the image for this Figure. (C) AT3G10845. Transposed gene is flanked by a direct repeat whose sequence occurs as a singlet in the A. lyrata orthologous site (top). Notice that the repeat sequence corresponds to the 3′ untranslated region of the gene adjacent to the transposed gene. (9.61 MB TIF) [file pgen.1000949.s001.tif]
